# CO_2_ Capture and Separation Properties in the Ionic Liquid 1-n-Butyl-3-Methylimidazolium Nonafluorobutylsulfonate

**DOI:** 10.3390/ma7053867

**Published:** 2014-05-14

**Authors:** Lingyun Zhou, Jing Fan, Xiaomin Shang

**Affiliations:** 1 School of Environment, Henan Normal University, Xinxiang 453007, Henan, China; E-Mails: lyzhou1980@163.com (L.Z.); shangxiaomin07@163.com (X.S.); 2 College of Resource and Environment, Henan Institute of Science and Technology, Xinxiang 453007, Henan, China

**Keywords:** carbon capture, selectivity, ionic liquid, Krichevsky-Kasarnovsky equation, 1-n-Butyl-3-methylimidazolium nonafluorobutylsulfonate

## Abstract

Recently, the use of ionic liquids (ILs) for carbon capture and separation processes has gained great interest by many researchers due to the high solubility of CO_2_ in ILs. In the present work, solubility measurements of CO_2_ in the novel IL 1-n-butyl-3-methylimidazolium nonafluorobutylsulfonate [C_4_mim][CF_3_CF_2_CF_2_CF_2_SO_3_] were performed with a high-pressure view-cell technique in the temperature range from 293.15 to 343.15 K and pressures up to about 4.2 MPa. For comparison, solubilities of H_2_, N_2_, and O_2_ in the IL were also measured at 323.15 K via the same procedure. The Krichevsky-Kasarnovsky equation was employed to correlate the measured solubility data. Henry’s law constants, enthalpies, and entropies of absorption for CO_2_ in the IL were also determined and presented. The CO_2_ solubility in this IL was compared with other ILs sharing the same cation. It was shown that the solubility of CO_2_ in these ILs follows the sequence: [C_4_mim][CF_3_CF_2_CF_2_CF_2_SO_3_] ≈ [C_4_mim][Tf_2_N] > [C_4_mim][CF_3_CF_2_CF_2_COO] > [C_4_mim][BF_4_], and the solubility selectivity of CO_2_ relative to O_2_, N_2_, and H_2_ in [C_4_mim][CF_3_CF_2_CF_2_CF_2_SO_3_] was 8, 16, and 22, respectively. Furthermore, this IL is regenerable and exhibits good stability. Therefore, the IL reported here would be a promising sorbent for CO_2_.

## 1. Introduction

The growing CO_2_ concentration in the atmosphere compels the scientific community to improve current carbon capture and storage (CCS) technologies, such as membrane-based separation, adsorption, and absorption [1–3]. For this purpose, researchers are extensively involved in the development of novel materials for energy and environmental applications. Among these materials, ionic liquids (ILs) have gained increasing interest because of their outstanding properties over traditional solvents, such as negligible vapor pressures, high thermal and chemical stability, strong solubility capacity, and good recyclability [4]. Among these particular properties, non-volatility potentially makes ILs “green” solvents, because ILs would not contaminate the atmosphere, even in small amounts. Also, tunability of their molecular structures and physicochemical properties are useful characteristics.

Because of these outstanding advantages, ILs have been applied in many research fields, such as analytical chemistry [5], biochemistry [6], electrochemistry [7], catalysis [8], and separation technology [9]. Among these applications, CO_2_ separation from post-combustion gas streams attracted significant attention due to CO_2_’s widely-known contribution to the greenhouse effect. Over the past decades, Blanchard *et al.* [10,11], Anthony *et al.* [12,13], Shariati and Peters [14,15], and Kim *et al.* [16] made extensive contributions to this field. Many ILs, especially the imidazolium ILs, have been successfully synthesized and used for carbon capture [10–20].

In order to improve the solubility of CO_2_ in ILs, usually two approaches have been used to optimize the molecular structure of ILs. First, the imidazolium cation was modified by addition of branching chains or some polar groups, such as ether linkages [21] and nitriles [22], to increase the free volume for enhanced CO_2_ occupation. Second, a functional CO_2_-philic group or anion was introduced by fluorination, carbonylation, or sulfonation to the cation to stabilize the surrounding CO_2_ [20,23–25]. Additionally, it was found that the nature of the anions of ILs play a larger role in the solubility of CO_2_ than the cations [26–28]. For example, the IL containing highly fluorinated anions, Tris(pentafluoroethyl)trifluorophosphate [FAP^−^], was proved to have the highest CO_2_ solubility among the ILs with the same cations [29]. Furthermore, C-F bonds were found to have several unique properties, such as increased rigidity and decreased polarity [30]. These properties not only lead to higher gas solubility in highly fluorinated ILs, but also simplify the regeneration of the IL.

The above merits inspired us to combine the two classes of CO_2_-philic functional groups, *i.e.*, sulfonation and fluorination phases, together with increasing the C–F bonds to a certain extent to produce the IL 1-n-butyl-3-methylimidazolium nonafluorobutyl sulfonate [C_4_mim][CF_3_CF_2_CF_2_CF_2_SO_3_], which was expected to exhibit a higher solubility for CO_2_ and an improved energy-saving regeneration. To the best of our knowledge, this is the first time that [C_4_mim][CF_3_CF_2_CF_2_CF_2_SO_3_] is used for carbon capture.

In addition to absorption performance and recyclability, the selectivity of ILs is of great importance. Highly efficient separation of CO_2_ from other “light” gases (O_2_, N_2_, H_2_, and hydrocarbons) is also a major issue in the CO_2_ capture from flue gases. Therefore, the ideal gas solubilities of N_2_, O_2_, H_2_, and CO_2_ in the IL were measured in this work. The mole fraction solubility of a single gas in [C_4_mim][CF_3_CF_2_CF_2_CF_2_SO_3_] was expressed as Henry’s constant, as deduced from the Krichevsky-Kasarnovsky equation. For each gas pair of CO_2_ to the three other gases tested, the ideal solubility selectivity was calculated via Henry’s constants to determine if the IL displayed a good ideal gas separation performance. The thermodynamic properties of CO_2_ in [C_4_mim][CF_3_CF_2_CF_2_CF_2_SO_3_] were also estimated. Additionally, a comparison was made for the solubilities of CO_2_ in the studied IL and in the homologues of imidazolium salts to determine the anionic effect.

## 2. Results and Discussion

### 2.1. Experimental Solubilities and Anionic Effects of the ILs

**Figure 1.**
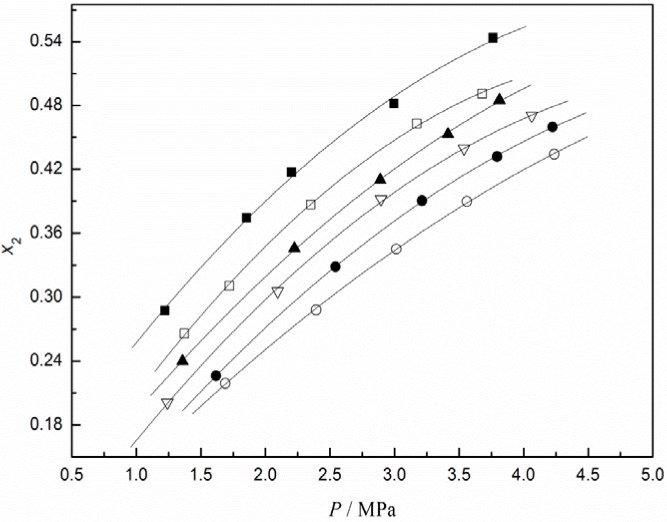
CO_2_ solubilities in [C_4_mim][CF_3_CF_2_CF_2_CF_2_SO_3_] as a function of pressure at different temperatures: (■, 293.13 K; □, 303.15 K; ▲, 313.15 K;▽, 323.15 K; •, 313.15 K; ○, 343.15 K). Lines were calculated via the Krichevsky-Kasarnovsky equation.

In this work, the experimental solubility of CO_2_ in [C_4_mim][CF_3_CF_2_CF_2_CF_2_SO_3_] was measured in the temperature range from 293.15 to 343.15 K and pressures up to about 4.2 MPa, and the results are shown in Table 1. The same measurements for N_2_, O_2_, and H_2_ were determined at 323.15 K and the results are given in Table 2. Among these experimental data, CO_2_ solubilities in [C_4_mim][CF_3_CF_2_CF_2_CF_2_SO_3_] are graphically presented in Figure 1 as a function of pressure at different temperatures. The results in Figure 1 show that either a decrease in temperature or an increase in pressure leads to an increase in CO_2_ solubility, as typically expected from gas solubility in liquids.

**Figure 2.**
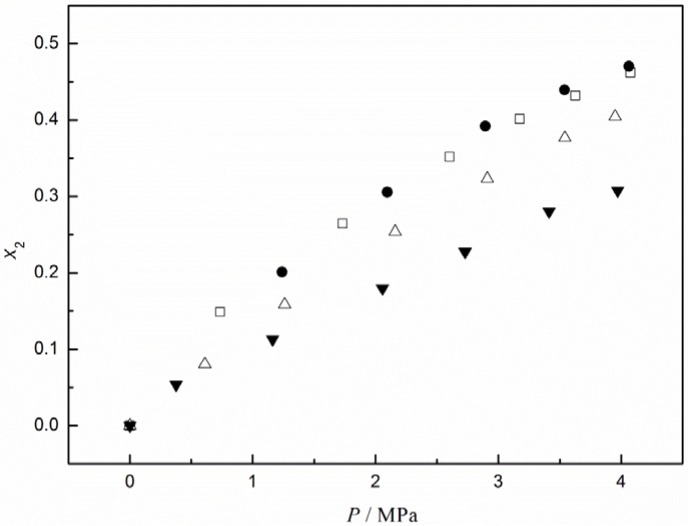
Comparison of CO_2_ solubility in different ionic liquids at T = 323.15 K: (•, [C_4_mim][CF_3_CF_2_CF_2_CF_2_SO_3_]), this work; (□, [C_4_mim][TF_2_N]), [31]; (△, [C_4_mim][CF_3_CF_2_CF_2_COO]), [31]; (▼, [C_4_mim][BF_4_]), [31]. Reproduced with permission from [31]. Copyright 2013 Elsevier.

To better understand the absorption performance of CO_2_ in [C_4_mim][CF_3_CF_2_CF_2_CF_2_SO_3_], we compared CO_2_ solubilities in this IL to those in other ILs containing the same cation, as reported in our recent work [31]. These were 1-n-butyl-3-methylimidazolium bis(trifluoromethyl)sulfonylimide ([C_4_mim][TF_2_N]), 1-n-butyl-3-methylimidazolium heptafluorobutyrate ([C_4_mim][CF_3_CF_2_CF_2_COO]), and 1-n-butyl-3-methylimidazolium terafluoroborate ([C_4_mim][BF_4_]). The isotherms of CO_2_ in these ILs at 323.15 K are plotted in Figure 2. It can be seen that [C_4_mim][CF_3_CF_2_CF_2_CF_2_SO_3_] and [C_4_mim][TF_2_N] basically exhibited the same solubility values for CO_2_, although those in [C_4_mim][CF_3_CF_2_CF_2_CF_2_SO_3_] were slightly greater at higher pressures. The solubility of CO_2_ in [C_4_mim][CF_3_CF_2_CF_2_COO] was somewhat lower than its solubility in [C_4_mim][CF_3_CF_2_CF_2_CF_2_SO_3_]. These results suggest that the good capacity of [C_4_mim][CF_3_CF_2_CF_2_CF_2_SO_3_] and [C_4_mim][TF_2_N] for CO_2_ adsorption may be attributed to the combination of fluorous alkyl chains and S=O bonds. Also shown in Figure 2 is the solubility of CO_2_ in [C_4_mim][BF_4_], which is generally used as a reference, possessing moderate absorption performance among the imidazolium-based ILs. Clearly, the absorption capacity of CO_2_ in the other three ILs is larger than in [C_4_mim][BF_4_].

### 2.2. Correlation of the Experimental Data via the Krichevsky-Kasarnovsky Equation

The Krichevsky-Kasarnovsky equation has been widely applied to calculate the solubility of gases in liquid solvents up to high pressures [32–35]. This equation can be described as follows [36]:

$$\text{ln}\frac{f_{2}(T,P)}{x_{2}}=\text{ln}H_{2}^{P_{1}^{S}}+\frac{{\bar{V}}_{2}^{\infty }(P-P_{1}^{S})}{RT}$$ (1)

where *f_2_*(*T*, *P*) is the fugacity of gas solute 2 in the gas phase at pressure *P* and temperature *T; x*_2_ is the mole fraction of the gas dissolved in the liquid solvents; $P_{1}^{S}$ is the saturated vapor pressure of liquid solvents; ${\bar{V}}_{2}^{\infty }$ is the partial molar volume of gas at infinite dilution of the liquid solvents; $H_{2}^{P_{1}^{S}}$ is Henry’s constant of gas in the liquid solvents at pressure $P_{1}^{S}$, and *R* is the gas constant. Since the vapor pressure of ILs is negligible, the fugacity of the gas in the gas-IL systems, *f*_2_(*T*, *P*), can be substituted for the pure gaseous phase. Thus, the saturated vapor pressure of ionic liquid $P_{1}^{S}$ can be considered to be zero. Therefore, Equation (1) can be expressed as:

$$\text{ln}\frac{f_{2}(T,P)}{x_{2}}=\text{ln}H_{2}+\frac{{\bar{V}}_{2}^{\infty }}{RT}P$$ (2)

The fugacity of pure gas *f*_2_(*T*, *P*) can be obtained according to the following equation:

$$f_{2}\left(T,P\right)=\phi \left(T,P\right)P$$ (3)

in which ϕ is the fugacity coefficient at pressure *P* and temperature *T*, and can be evaluated via the Soave–Redlich–Kwong (SRK) equation of state [37]:

$$P=\frac{RT}{v-b}-\frac{a}{v(v+b)}$$ (4)

$$a=0.42747\left(\frac{R^{2}T_{c}^{2}}{P_{c}}\right)\alpha \left(T\right)$$ (5)

$$\alpha \left(T\right)=1+\beta \left[1-{\left(\frac{T}{T_{c}}\right)}^{0.5}\right]$$ (6)

$$\beta =0.480+1.574\omega -0.176\omega^{2}$$ (7)

$$b=\frac{0.08664RT}{P_{c}}$$ (8)

$$\text{ln}\phi =Z-1-\text{ln}\left[Z\left(1-\frac{b}{v}\right)\right]-\frac{a\alpha }{bRT}\text{ln}\left(1+\frac{b}{v}\right)$$ (9)

where *P* is the pressure; *T* is the temperature; *a* and *b* are the constants of the SRK equation of state; *v* is the molar volume; *T*_c_ is the critical temperature and *P*_c_ is the critical pressure; α(*T*) is a temperature-dependent parameter; ω is the acentric factor; and *Z* is the compressibility factor.

**Figure 3.**
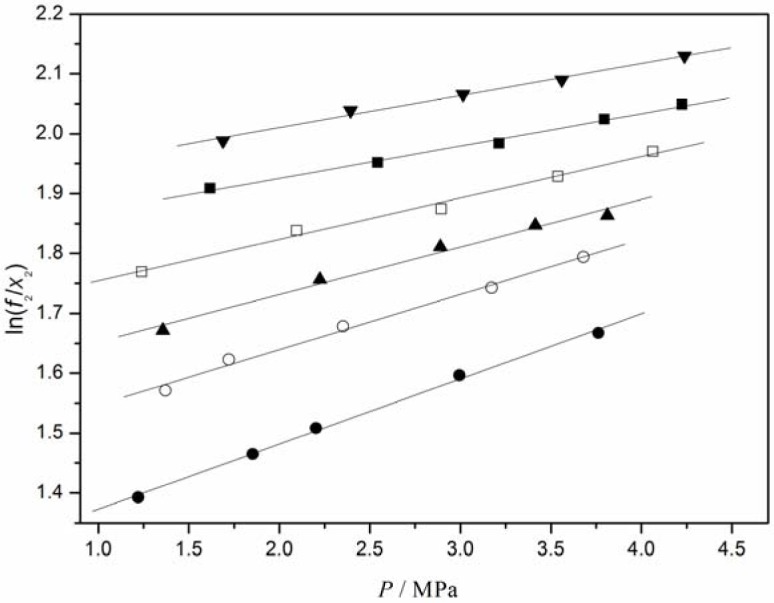
ln(*f*_CO2_*/x*) as a function of pressure at different temperatures: (•, 293.13 K; ○, 303.13 K; ▲, 313.15 K;□, 323.15 K; ■, 333.15 K; ▼, 343.15 K), this work; The lines were calculated via linear regression.

The experimental solubility data in the CO_2_/[C_4_mim][CF_3_CF_2_CF_2_CF_2_SO_3_] system were correlated with the Krichevsky-Kasarnovsky equation as a function of pressure at different temperatures. For the sake of this, Henry’s constants and partial molar volumes of CO_2_ at different temperatures should be first obtained. Based on Equation (2), the plot of ln(*f*_2_/*x*_2_) versus *P* is given in Figure 3, from which Henry’s constant and the partial molar volume of CO_2_ at a given temperature were calculated from the intercept and slope of the plot, respectively. At the same time, Henry’s constants for O_2_, N_2_, and H_2_ in [C_4_mim][CF_3_CF_2_CF_2_CF_2_SO_3_] were also calculated from the experimental solubility data by the same procedure. The obtained values for O_2_, N_2_, H_2_, and CO_2_ are listed in Table 3.

**Figure 4.**
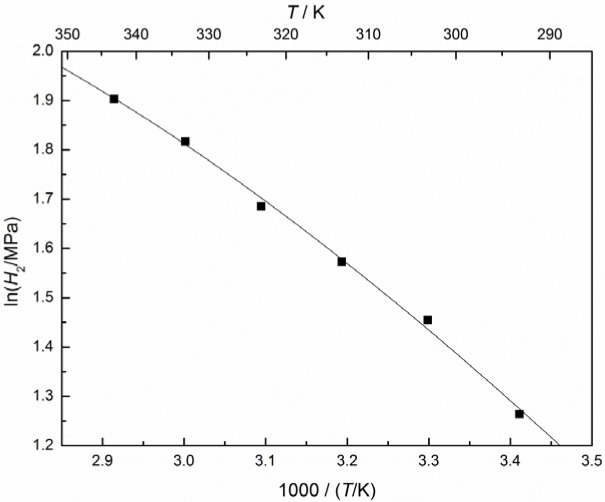
Henry’s constants for CO_2_ in [C_4_mim][CF_3_CF_2_CF_2_CF_2_SO_3_] at zero pressure and mole fraction scale as a function of the inverse temperature: ■, extrapolated experimental values; –, correlation results.

From the values of Henry’s constants for CO_2_ in the IL at different temperatures, the temperature dependence of Henry’s constant can be expressed as:

$$\text{ln}(H_{2}/\text{MPa})=14.7212-2709.54/(T/K)-0.001434(T/K)$$ (10)

and the plot of ln*H_2_ versus T* or 1/*T* is given in Figure 4. The final results for the partial molar volume can be correlated via:

$$V_{2}^{\infty }/{\text{cm}}^{3}\cdot {\text{mol}}^{-1}=453.73-0.9678(T/K)+0.0014{\left(T/K\right)}^{2}$$ (11)

Hence, the experimental solubility values for CO_2_ in [C_4_mim][CF_3_CF_2_CF_2_CF_2_SO_3_] at a given temperature and different pressures can be correlated via the Krichevsky-Kasarnovsky equation with Henry’s constants given by Equation (10) and the partial molar volume given by Equation (11). The consistency of the obtained correlation values with the experimental solubility data for CO_2_ in this IL may be assessed by the average of relative deviation, ARD, defined by:

$$ARD=\frac{1}{n}\sum_{i=1}^{1}\left|\frac{x_{2,i}^{\text{model}}-x_{2,i}}{x_{2,i}}\right|$$ (12)

where *x*_2,i_ is the experimental solubility of CO_2_ in [C_4_mim][CF_3_CF_2_CF_2_CF_2_SO_3_] in terms of mole fraction and $x_{2,i}^{\text{model}}$ is the corresponding value correlated via the Krichevsky-Kasarnovsky equation. The resulting ARD was found to be 0.69%, which is small enough to show that the Krichevsky-Kasarnovsky equation was suitable to describe the solubility behavior of CO_2_ with high accuracy in the investigated system.

### 2.3. Solubility Selectivity and Solution Thermodynamic Properties of CO_2_ in the IL

In order to determine the ideal gas separation performance of [C_4_mim][CF_3_CF_2_CF_2_CF_2_SO_3_], we compared the values of Henry’s law constants for H_2_, N_2_, O_2_, and CO_2_ in the IL at 323.15 K (Table 3), and then calculated the ideal solubility selectivity of the gas pairs. As generally known, a low value of Henry’s law constant indicates a high gas solubility and vice versa. From Table 3, it can be seen that at the same temperature, the magnitude of Henry’s constants for the gases in the IL decreases in the order: H_2_ > O_2_ > N_2_ > CO_2_, which indicates that the solubility of these gases in the IL follows the sequence: CO_2_ > O_2_ > N_2_ > H_2_. The solubility selectivity of [C_4_mim][CF_3_CF_2_CF_2_CF_2_SO_3_] for CO_2_/O_2_, CO_2_/N_2_ and CO_2_/H_2_ was calculated to be 8, 16, and 22, respectively, which is consistent with the results reported for the other fluorinated ILs in the literature [38].

To examine the reasons why this IL has different absorption performances for these gases, we determined the interactions between the IL and the gas molecules. For comparison, some important structural properties for CO_2_, O_2_, N_2_, and H_2_, such as polarizability, dipole moment, and quadrupole moment [39,40], are listed in Table 4. These data clearly indicate that the value of the quadrupole moment of CO_2_ is much higher than that of the other three gases. Therefore, we postulated that the higher solubility of CO_2_ in the IL was due to the larger quadrupole moment. Additionally, as mentioned earlier, the good solubility of CO_2_ in [C_4_mim][CF_3_CF_2_CF_2_SO_3_] could also be ascribed to fluorination and the presence of S=O bonds. On the contrary, the lowest solubility of H_2_ in the IL was governed solely by its lowest polarizability. Yet, the fact that O_2_ exhibited a higher solubility in the IL than N_2_ seems to show that both quadrupole moment and polarizability do not play determinant role in their solubility performances. The behavior of O_2_ in the IL might very likely be attributed to the affinity of fluorocarbons for O_2_ [41]. Compared to O_2_, N_2_, and H_2_, the significantly higher solubility of CO_2_ suggests that it should be possible to capture CO_2_ from flue gases.

Additionally, we calculated the thermodynamic properties for the adsorption of CO_2_ in [C_4_mim][CF_3_CF_2_CF_2_CF_2_SO_3_] using the data of Henry’s constants. The partial molar enthalpy and entropy at a specific pressure can be calculated from the following thermodynamic relationships [27]:

$$\Delta H=R{\left(\frac{\partial \text{ln }H}{\partial (1/T)}\right)}_{P}$$ (13)

$$\Delta S=R{\left(\frac{\partial \text{ln }H}{\partial (\text{ln }T)}\right)}_{P}$$ (14)

It was found that within the investigated pressure range, the calculated values for ∆*H* and ∆*S* were weakly dependent on the pressure and nearly equaled to the values at infinite dilution, as determined via the van ’t Hoff equation [27]. From this approximation and aforementioned equations, we obtained values of −11.9 kJ·mol^−1^ for the enthalpy and −40.0 J·mol^−1^·K^−1^ for the entropy of CO_2_ absorption in [C_4_mim][CF_3_CF_2_CF_2_CF_2_SO_3_]. These values are very close to those reported for CO_2_ in [C_4_mim][TF_2_N], *i.e.*, −12.5 kJ·mol^−1^ for the enthalpy and −41.3 J·mol^−1^·K^−1^ for the entropy. This indicates that almost the same strong interactions and structural ordering occurred in these two systems. Considering the fact that these two ILs have the same affinity groups of fluorous alkyl chains and S=O bonds for CO_2_, suggested that there would be little difference in the solubilities of CO_2_ in these two ILs. This was confirmed by direct comparison of the solubility values of CO_2_ in [C_4_mim][CF_3_CF_2_CF_2_CF_2_SO_3_] and [C_4_mim][Tf_2_N], as shown in Figure 2.

In addition, a comparison was made for the heat of absorption of CO_2_ in [C_4_mim][CF_3_CF_2_CF_2_CF_2_SO_3_] and in non-IL solvents. It was found that the enthalpy value of CO_2_ dissolved in the IL is similar to those of CO_2_ in conventional organic solvents, such as heptane and ethanol, where physical absorption was observed with absorption enthalpies in the range from −9.7 to −12.8 kJ·mol^−1^ [12]. This suggests that the absorption of CO_2_ in [C_4_mim][CF_3_CF_2_CF_2_CF_2_SO_3_] is mainly physical absorption in nature, although the ATR-IR study of Kazarian and co-workers [42] indicated possible chemical interactions between the anion and CO_2_.

### 2.4. Recyclability and Reuse of the IL

**Figure 5.**
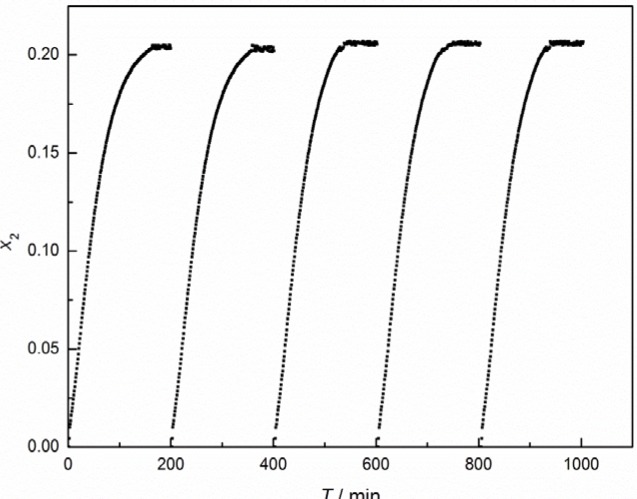
CO_2_ absorption-desorption cycles in [C_4_mim][CF_3_CF_2_CF_2_CF_2_SO_3_].

To evaluate recyclability of the IL, the determination of the absorption capacity of [C_4_mim][CF_3_CF_2_CF_2_CF_2_SO_3_] for CO_2_ was performed by recycling five times at 323 K. The sample cell containing the IL was pressurized with CO_2_ to 1.70 MPa with tiny pressure fluctuations within the five measurements. The pressure fall and corresponding time were monitored and recorded until equilibrium. The amount of dissolved CO_2_ in the IL at given time points was calculated on the basis of a pressure-decay observation using the SRK equation of state [25]. In order to explore an energy-efficient method to regenerate the IL, we tried to regenerate the CO_2_-saturated IL in two ways. One desorption experiment was performed at 323 K for 1 h by flushing with N_2_ at 20 mL·min^−1^, and, alternatively, by degassing the equilibrium cell at 323 K for 2 h. It was shown that both of the regeneration ways were very efficient. The CO_2_ absorption-desorption cycles in [C_4_mim][CF_3_CF_2_CF_2_CF_2_SO_3_] by degassing are depicted in Figure 5. It can be seen that the absorption capacities of [C_4_mim][CF_3_CF_2_CF_2_CF_2_SO_3_] for CO_2_ were quite constant in the five absorption-desorption cycles. Additionally, ^1^H and ^13^C NMR studies were conducted for the regenerated IL, and the results showed that no new compounds could be observed during the CO_2_ absorption and desorption processes.

## 3. Experimental Section

### 3.1. Materials

The ionic liquid [C_4_mim][CF_3_CF_2_CF_2_CF_2_SO_3_] was obtained from Shanghai Cheng Jie Chem. Co. Ltd (Shanghai, China), with the stated purity no less than 99%. The purchased IL were first dried under vacuum at 323 K for at least 48h and then stored in a vacuum drier before use to prevent it from absorbing water. CO_2_, H_2_, N_2_, and O_2_ were purchased from Beijing Paraxair Utility Gas Ltd Co. (Beijing, China) with mass purities of 99.995%, 99.999%, 99.99% and 99.95%, respectively. These gases were used in the experiments without further purification.

### 3.2. Solubility Experimental Apparatus and Procedure

**Figure 6.**
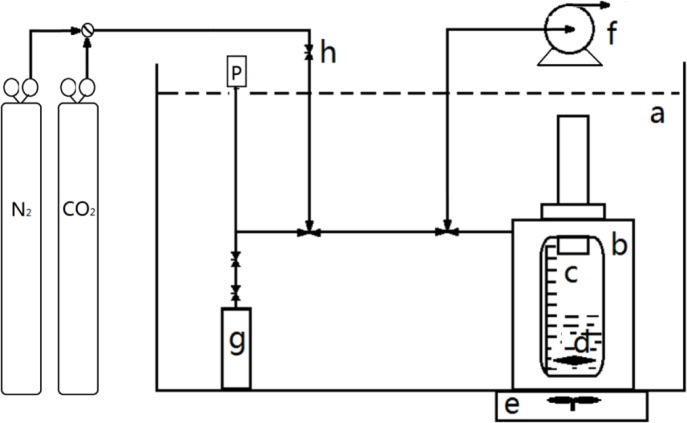
Apparatus for the solubility measurements: a, water bath; b, high-pressure cell; c, volume scale; d, magnetic stirring bar; e, magnetic stirrer; f, vacuum pump; g, gas storage tank; h, needle valve; P, pressure gauge.

The detailed experimental apparatus and procedure for the solubility measurements were described previously [31]. The apparatus is schematically depicted in Figure 6. Before the solubility measurements, the volume of the gas tank and total apparatus was determined. In a typical experiment, a known mass of the IL was loaded in the high pressure optical cell, which was placed in a transparent water bath with a magnetic stirrer. The desired temperature was controlled by a temperature controller. Subsequently, the sample cell was evacuated to 10^−9^ bar for 12 h at 323 K to remove dissolved gases and water. After the valve was opened, high pressure gas from the gas storage tank was transferred to the cell for absorption by the IL. Pressure in the system dropped gradually, as observed on the pressure gauge connected to the solubility experimental apparatus. When the pressure remained stable for 1 h, the system was considered to reach a state of equilibrium. The volume of the gas phase at equilibrium could be calculated based on the volume of the IL (volume scale on the optical cell). The gas density of the gas phase in the equilibrated system was determined from the gas mass in the gas tank at equilibrium divided by the volume of the gas tank. Based on the above parameters, the mass of the gas in the gas phase at equilibrium was determined and consequently the gas mass absorbed by the IL.

The mass of gas and IL was determined with an electronic balance with a precision of ±0.0001 g. The temperature of the solubility experiments controlled by a temperature controller was within an uncertainty of ±0.1 K. Gas pressures with a precision of ±0.001 MPa were measured with a pressure gauge in the range from 0 to 10 MPa. The IL volume read from the volume scale on the optical cell was within the precision of ±0.02 mL. All data shown in the tables and figures were the average of at least three trials. From the error analysis, the estimated uncertainty in the solubility is ±0.8%.

## 4. Conclusions

In this work, we reported the solubilities of CO_2_, O_2_, N_2_, and H_2_ in [C_4_mim][CF_3_CF_2_CF_2_CF_2_SO_3_] and compared the CO_2_ solubility in this IL to other 1-butyl-3-methylimidazolium-based ILs. It was found that [C_4_mim][CF_3_CF_2_CF_2_CF_2_SO_3_] has a comparable ability for CO_2_ capture to [C_4_mim][TF_2_N] due to a combination of fluorination and the presence of S=O bonds. By using the Krichevsky-Kasarnovsky equation, Henry’s constants of CO_2_, O_2_, N_2_, and H_2_ in the IL were determined and solution thermodynamic properties for IL/CO_2_ system were derived. The solubility selectivity of [C_4_mim][CF_3_CF_2_CF_2_CF_2_SO_3_] for each gas pair tested was calculated from their corresponding Henry’s constants. It was shown that the values for CO_2_/O_2_, CO_2_/N_2_, and CO_2_/H_2_ were 8, 16, and 22, respectively. Also, the large affinity of the IL for CO_2_ was explained by more favorable interactions between the gas and the IL molecules. In addition, the gas solubility expressed as mole fraction at given temperature and pressure was correlated via the Krichevsky-Kasarnovsky equation with an average relative deviation of about 0.69% for the system of CO_2_/[C_4_mim][CF_3_CF_2_CF_2_CF_2_SO_3_]. We estimate that the present study will aid in the design of promising absorbents for CO_2_ by estimating both the absorption capacity and the selectivity.

## Figures and Tables

**Table 1.** Experimental solubility values, expressed as the mole fraction *x_2_* of CO_2_ in [C_4_mim][CF_3_CF_2_CF_2_CF_2_SO_3_] at temperature *T* as a function of pressure *P*.

*P* (MPa)	*x*_2_	*P* (MPa)	*x*_2_	*P* (MPa)	*x*_2_

***T* = 293.15 K**		***T* = 303.15 K**		***T* = 313.15 K**	
1.221	0.2831	1.371	0.2659	1.357	0.2399
1.853	0.3837	1.721	0.3128	2.224	0.3458
2.202	0.4261	2.352	0.3867	2.889	0.4101
2.995	0.4989	3.173	0.4631	3.413	0.4529
3.763	0.5436	3.679	0.5022	3.812	0.4851

***T* = 323.15 K**		***T* = 333.15 K**		***T* = 343.15 K**	

1.240	0.2012	1.617	0.2262	1.689	0.2190
2.095	0.3056	2.543	0.3285	2.393	0.2880
2.894	0.3921	3.213	0.3907	3.014	0.3452
3.538	0.4396	3.795	0.4319	3.560	0.3900
4.063	0.4705	4.224	0.4598	4.238	0.4344

**Table 2.** Experimental solubility values, expressed as the mole fraction *x_2_* of N_2_, O_2_, and H_2_ in [C_4_mim][CF_3_CF_2_CF_2_CF_2_SO_3_] at 323.15 K as a function of pressure *P*.

O_2_		N_2_		H_2_	

*P* (MPa)	*x*_2_	*P* (MPa)	*x*_2_	*P* (MPa)	*x*_2_
2.243	0.0458	2.355	0.0272	2.296	0.0176
4.027	0.0789	4.384	0.0495	4.283	0.02785
5.619	0.1035	5.876	0.0657	5.694	0.0336
6.705	0.1172	6.838	0.0757	6.804	0.03943
8.131	0.1362	8.169	0.0899	7.844	0.04250

**Table 3.** Henry’s constants *H*_2_ for CO_2_, O_2_, N_2_, and H_2_ in [C_4_mim][CF_3_CF_2_CF_2_CF_2_SO_3_] at temperature *T* and zero pressure ^a^.

Gas	T (K)	*H*_2_ ± σ (MPa)
CO_2_	293.15	3.54 ± 0.03
	303.15	4.39 ± 0.03
	313.15	4.82 ± 0.04
	323.15	5.39 ± 0.03
	333.15	6.16 ± 0.03
	343.15	6.71 ± 0.04

O_2_	323.15	44.8 ± 0.8

N_2_	323.15	84.8 ± 7

H_2_	323.15	116 ± 4

a on the mole fraction scale; σ is the standard deviation.

**Table 4.** Polarizability (α); dipole moment (μ); and quadrupole moment (*Q*) for CO_2_, N_2_, O_2_, and H_2_. Reproduced with permission from [39,40]. Copyright 1999, Prentice Hall PTR and Copyright 1966, Taylor and Francis Group.

Gas	α × 10^24^ (cm^3^)	μ × 10^18^ (esu·cm)	*Q* × 10^26^ (esu·cm^2^)
CO_2_	2.64	0	4.3
O_2_	1.60	0	0.39
N_2_	1.74	0	1.5
H_2_	0.81	0	0.662
